# Pseudo-Spectral Spatial Feature Extraction and Enhanced Fusion Image for Efficient Meter-Sized Lunar Impact Crater Automatic Detection in Digital Orthophoto Map

**DOI:** 10.3390/s24165206

**Published:** 2024-08-11

**Authors:** Huiwen Liu, Ying-Bo Lu, Li Zhang, Fangchao Liu, You Tian, Hailong Du, Junsheng Yao, Zi Yu, Duyi Li, Xuemai Lin

**Affiliations:** 1 School of Space Science and Physics, Institute of Space Sciences, Shandong University, Weihai 264209, China; huiwenliu@mail.sdu.edu.cn (H.L.); liufc@mail.sdu.edu.cn (F.L.); youtian@mail.sdu.edu.cn (Y.T.); duhailong@mail.sdu.edu.cn (H.D.); maimai@mail.sdu.edu.cn (X.L.); 2 National Space Science Data Center, National Space Science Center, Chinese Academy of Sciences, Beijing 100190, China; 3 School of Mechanical, Electrical and Information Engineering, Institute of Mechanical, Shandong University, Weihai 264209, China; jsyao@mail.sdu.edu.cn (J.Y.); yuzi_sdu@163.com (Z.Y.); 4 SDU-ANU Joint Science College, Shandong University, Weihai 264209, China; liduyi2005@mail.sdu.edu.cn

**Keywords:** automatic crater detection, meter-sized crater, pseudo-spectral spatial feature extraction, YOLOv5, optimization of post-processing parameters

## Abstract

Impact craters are crucial for our understanding of planetary resources, geological ages, and the history of evolution. We designed a novel pseudo-spectral spatial feature extraction and enhanced fusion (PSEF) method with the YOLO network to address the problems encountered during the detection of the numerous and densely distributed meter-sized impact craters on the lunar surface. The illumination incidence edge features, isotropic edge features, and eigen frequency features are extracted by Sobel filtering, LoG filtering, and frequency domain bandpass filtering, respectively. Then, the PSEF images are created by pseudo-spectral spatial techniques to preserve additional details from the original DOM data. Moreover, we conducted experiments using the DES method to optimize the post-processing parameters of the models, thereby determining the parameter ranges for practical deployment. Compared with the Basal model, the PSEF model exhibited superior performance, as indicated by multiple measurement metrics, including the precision, recall, *F*_1_-score, *mAP*, and robustness, etc. Additionally, a statistical analysis of the error metrics of the predicted bounding boxes shows that the PSEF model performance is excellent in predicting the size, shape, and location of impact craters. These advancements offer a more accurate and consistent method to detect the meter-sized craters on planetary surfaces, providing crucial support for the exploration and study of celestial bodies in our solar system.

## 1. Introduction

Impact craters are the most prominent and universally significant geological features on planetary surfaces [1]. Impact craters offer vital clues for the evaluation of planetary resources, such as the subsurface minerals and water resources. For example, Dhingra et al. proposed that we can reveal the mineral resources in the lunar subsurface by analyzing the impact melt deposits at the bottom of impact craters [2,3]. Crawford et al. reported that due to the low rotational inclination (1.5°) of the Moon, the bottom of impact craters near the poles remains permanently shadowed, potentially stabilizing the deposits of water ice [4]. Meanwhile, the distribution and characteristics of impact craters are pivotal in determining the geological ages of celestial bodies and in decoding their geological evolutionary histories, such as the Moon and Mars. For instance, Yue et al. utilized the crater size–frequency distribution to date planetary surfaces, emphasizing its essential role in formulating models of lunar regolith formation and deducing the thicknesses of the regolith and basalt layers [5]. Moreover, in planetary exploration missions, the spatial distribution of impact craters is particularly critical for choosing probe landing sites [6] and developing rover routes [7,8], which need to avoid rugged terrain, thereby ensuring the safety and efficiency of exploration missions. For instance, Menon et al. noted that the horizons created by impact craters may impede power generation and communication for lunar landers and rovers, while Hu et al. highlighted that global path planning algorithms from orbital remote sensing data can pre-plan lunar rover routes, enhancing the exploration efficiency and system safety [9,10].

Lunar surface research has revealed a vast number of impact craters, which are crucial for understanding lunar and planetary evolution. Robbins established a database with 2 million lunar impact craters, including 1.3 million craters exceeding 1 km in diameter [11]. Efficient and precise automatic crater detection algorithms (CDAs) make it easier to create the comprehensive databases of impact craters on the planetary surface [12]. Using the Chang’E data and transfer learning in deep neural networks, Yang et al. developed an automatic CDA to build a new database containing 117,240 lunar impact craters with diameters over 1 km [13]. Then, they analyzed the stratigraphic coverage relationships, crater morphology, optical maturity, cumulative size–frequency distributions, and thermophysical characteristics via this new database. Fairweather et al. developed an automatic CDA using a modified YOLOv3 framework, which can detect craters as small as 20 m in diameter and analyze images taken at various incident light angles and stages of degradation [12]. Yang et al. introduced a comprehensive and effective deep learning workflow for lunar crater detection using digital elevation model (DEM) data and then assessed three popular detection frameworks across nine CNN models [14].

Up to now, algorithms to automatic identify large-scale impact craters have been well developed. However, an automatic CDA for meter-sized impact craters is still lacking [15]. In this work, meter-sized impact craters are defined as impact craters less than 10 pixels (about 14 m for the data we used) in diameter. Some investigations showed that the size–frequency distribution of craters follows a power–law relationship [16]. Kawashima et al. noted that near Whipple Crater, the cumulative crater frequency (CCF) for 10 m craters is approximately 60 km^−2^, which is 200 times that of 100 m craters (approximately 3 × 10^−1^ km^−2^), and it is also 20,000 times that of 1000 m craters (approximately 3 × 10^−3^ km^−2^) [17]. Thus, the manual recognition of these massive meter-sized craters is labor-intensive [18]. With respect to the automatic CDA, the sparse features in meter-sized craters pose challenges for feature learning in deep neural networks [19]. In the general deep learning frameworks, the process of multiple downsampling weakens the ability to resolve the fine details of targets [20,21,22,23]. Therefore, identification of meter-sized impact craters is frequently realized using the landing camera (LCAM) of planetary surface landers, which captures high-resolution images during the descent and landing processes [10,24]. However, the scope of images captured by the LCAM is restricted to areas surrounding the landing sites. The identification of meter-sized impact craters on planetary surfaces in larger areas still relies on remote sensing images taken from orbiters.

Thus, lots of studies have been carried out to obtain the sparse features of small craters to improve the identification ability of deep learning algorithms. For instance, Grassa et al. utilized super-resolution images with the YOLO framework to automatically detect craters using images taken by the Lunar Reconnaissance Orbiter Narrow Angle Camera (LRO-NAC), while Tewari et al. developed an arbitrary scale super-resolution technique to amplify the features of small impact craters in LRO-NAC images [25,26]. Additionally, there are CDA developed based on multi-stage neural networks such as R-CNN [27]. For instance, Zang et al. developed Crater R-CNN with TTSN, which demonstrated strong robustness and good generalization ability for detecting impact craters ranging from 100 to 1000 m in diameter in both the Maria and Highland regions of the Moon [28]. Prete et al. developed a CDA using Cascade Mask R-CNN to successfully detect larger impact craters in both the polar and equatorial regions of the Moon, but it still faced challenges in identifying small-sized craters [29]. Chatterjee et al. demonstrated that YOLO offers high accuracy while being faster than comparable CNNs such as R-CNN and Faster R-CNN [30]. In remote sensing images on the Moon, meter-sized impact craters usually lack features, equivalent to a typical Small Object Detection (SOD) [19] challenge. Although YOLO is extensively utilized for the detection of impact craters [25] and various other terrain elements on planetary surfaces [31], comprehensive studies have demonstrated that it is still a challenge for the YOLO framework to detect the SOD, due to the large receptive field of the deep feature maps, which lacks adequate features for small object identification [32,33,34]. Therefore, enriching the input features remains a crucial problem for the YOLO algorithm to enhance its ability to identify meter-sized lunar impact craters. There is another method, i.e., the multichannel input method, that can introduce additional features to enrich the original image features. For instance, Mu et al. developed an automatic CDA that integrates multimodal data including Digital Orthophoto Map (DOM), DEM, and slope data, along with seven kinds of visualization stretching methods [35]. After extensive experiments, they demonstrated that DOM data are the most effective data to detect small impact craters. Meanwhile, automatic CDAs using edge detection techniques can extract lunar crater edge features by grayscale mutation on both sides of the impact crater rims [36]. For instance, Saheba et al. explored adaptive edge detection algorithms such as the Gaussian filter for lunar surface crater topology under different illuminations and terrains [37]. Additionally, Zuo et al. utilized a ternary method based on grayscale frequency statistics to extract highlighted and shaded areas of impact craters [38].

The automatic detection of meter-sized craters remains challenging due to their sparse features and limitations in current deep learning frameworks. Therefore, there is a need for a more efficient and accurate method to identify these small craters, particularly in large datasets. To address these questions for the automatic detection of meter-sized craters on the lunar surface via the YOLO algorithm, we proposed a pseudo-spectral spatial feature extraction and enhanced fusion (PSEF) image based on advanced edge detection and frequency analysis techniques to improve the detection accuracy. Based on DOM data, the illumination incidence features, isotropic features, and eigen frequency features are extracted by means of Sobel filtering, Laplacian of Gaussian (LoG) filtering, and frequency domain bandpass filtering, respectively. Then, the PSEF images are created by pseudo-spectral spatial techniques to preserve additional details from the original DOM data and address the disadvantage of feature scarcity in the detection of meter-sized lunar impact craters. This new proposed method significantly improves the detection performance of the YOLOv5 framework on meter-sized lunar impact craters with scarce features. Moreover, the method proposed in this paper offers an improved approach for the development of algorithms for other remote sensing SOD tasks, such as lunar rocks and Martian dunes.

## 2. Methodology

### 2.1. Data Source

**Figure 1:**
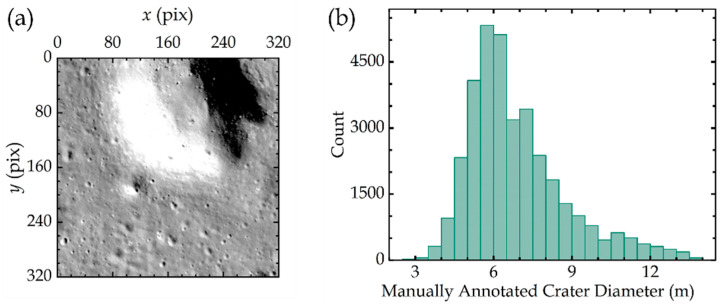
(**a**) An illustration of an impact crater slice. (**b**) The number–diameter distribution of 34,876 impact craters with diameters less than 14 m in our cropped 59 slices from the CE-4 landing site.

We used the DOM from LRO-NAC with the ID M1303619844 to build our dataset in this work, which shows a resolution of 1.4 m/pixel and an image size of 7800 × 19627 pixels [39,40]. This image was captured over the Chang’e-4 (CE-4) landing site, i.e., in the longitude range from 177.29° to 177.80° and the latitude range from −46.22° to −45.31°. This DOM features superior resolution and an appropriate solar elevation angle, which facilitates the performance of the CDA. Naturally, the method proposed in this paper is not limited to this particular DOM. This image is cropped into 320 × 320 pixel slices to construct our training, validation, and test datasets. An illustration of a slice is shown in Figure 1a. We chose 59 slices randomly to annotate the craters with diameters less than 10 pixels (14 m), producing a dataset of 34,876 tags in total. All targets identifiable as impact craters by manual inspection with diameters less than 10 pixels are tagged. The size–frequency distribution of our annotated impact craters is shown in Figure 1b.

Among those 59 slices, 34 slices containing 19,192 annotated craters are designated for training the network, while 15 slices with 9304 annotated craters are used for validation. The training dataset is used to adjust the parameters of the model by the features and patterns annotated in this dataset. The validation dataset in this study not only helps in tuning hyperparameters and mitigating overfitting by assessing the performance of the model on a separate subset of images during the training phase but also contributes to optimizing the non-maximum suppression (NMS) parameters of the preliminary model after training. This optimization determines the range for selecting and adjusting the post-processing parameters during the actual deployment of the model. Furthermore, for the testing process of our trained CDA, 10 slices comprising 6384 annotated craters are employed to evaluate the accuracy and the generalization ability of our proposed PSEF method.

### 2.2. Feature Extraction and Enhanced Fusion Method

**Figure 2:**
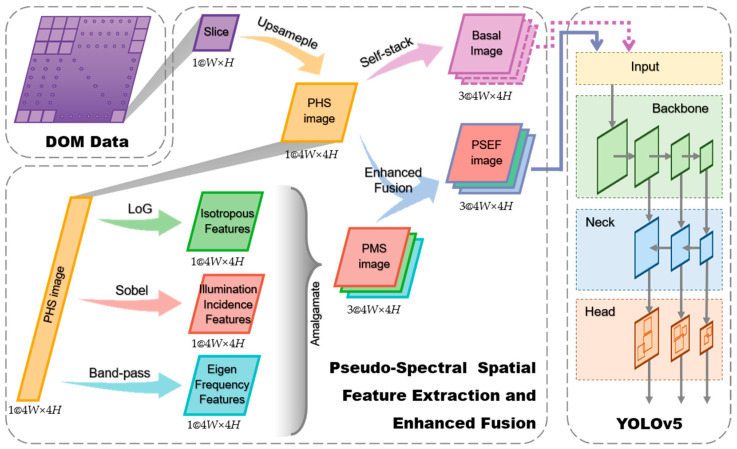
Schematic diagram of the PSEF method for detecting meter-sized impact craters. The parameter preceding the ‘@’ represents the number of channels in the image. The parameters *W* and *H* following ‘@’ denote the width and height of the image, respectively. In this figure, *W* and *H* are both set to 320 pixels, e.g., ‘1@4*W*×4*H*’ under the PHS image indicates that the PHS image is single-channel with a size of 1280 × 1280 pixels. The grey arrows indicate that the images connected by these arrows represent the same image at different stages of the process.

We proposed a pseudo-spectral spatial feature extraction and enhanced fusion method to encompass different characteristic aspects of these meter-sized craters. The flowchart of this approach is shown in Figure 2.

The grayscale DOM image is initially cropped into several slices. Each slice is then upsampled by bilinear interpolation to form a pseudo-hyperspectral (PHS) image. The PHS images with promoted resolution serve as one of the inputs in the subsequent enhanced fusion operations, which increases the operational flexibility in the following extraction processes, such as adjusting the convolution kernel sizes.

In the feature extraction stage for various modalities of meter-sized impact craters, the LoG operator [41], Sobel operator [42], and Fourier transform [43] are utilized to extract the isotropic edge features, illumination incidence features, and eigen frequency features, respectively. These three extracted features will then be integrated into a pseudo-multispectral (PMS) image of meter-sized impact craters from those three distinct modalities.

The Sobel operator is used to extract the edge information of impact craters from the input grayscale PHS images. As is well known, the shadows of all craters in one image are all in the same direction, whereas the domes or rocks exhibit the opposite shadow directions at the same time [28]. This feature of shadow direction is easily distinguishable during manual annotation. However, in deep learning networks such as YOLO, when the rotational transformations are used in data augmentation, it is challenging to distinguish the positive terrain features (e.g., the domes, rocks) and the negative terrain features (e.g., craters). Therefore, the angle of incident light is a crucial factor that needs careful consideration and effective utilization in the analysis of DOM data. The Sobel operator is a directional first-order differential operator that exhibits high sensitivity to the edge variations in specific directions [44]. When the direction of the Sobel operator is along the direction of the incident light, it will extract the features of this direction. The directional Sobel operator can effectively distinguish feature changes between the positive terrain features and the negative terrain features in the DOM data. Thus, the edge information of impact craters can be extracted from the input grayscale PHS images via the Sobel operator along the illumination direction, which is denoted as *M*. The output images *B*_0_ capturing the illumination incidence features is defined as Equation (1).

(1) $$B_{0}\left(x,y\right)=\sqrt{{\left[S\left(M\left(x,y\right),g=x\right)\right]}^{2}\cos\theta +{\left[S\left(M\left(x,y\right),g=y\right)\right]}^{2}\sin\theta }$$

where *S*(*M*(*x*, *y*), *g* = *x*) and *S*(*M*(*x*, *y*), *g* = *y*) are the Sobel operator calculated gradients in the horizontal and vertical directions, respectively. *θ* is the angle between the direction of incident light and the *x*-axis in the image.

The LoG operator is characterized as an isotropic second-order differential operator, which can effectively complement the directional first-order differential Sobel operator. By detecting zero-crossing points within the second derivative of the image [45], the LoG operator shows particular sensitivity to the edge continuity. Hence the LoG operator can accurately identify edge positions and integrate comprehensive contextual information from adjacent pixels [41,46]. The action of the LoG operator on the PHS image *M*(*x*, *y*) is defined as follows:

(2) $$G_{0}\left(x,y\right)=\sum_{i=-k}^{k}\sum_{j=-k}^{k}LoG\left(i,j,\sigma \right)\cdot M\left(x-i,\;y-j\right)$$

where *G*_0_(*x*, *y*) is the resulting image after applying the LoG operator to the input image *M* (*x* −*i*, *y* − *j*). Here, *i* and *j* represent the indices used to iterate over the pixels surrounding the LoG kernel, defining its application area on the image. The sums iterate over the range [*−k*, *k*], where *k* is the radius of the LoG kernel. The size of the kernel is determined by *σ*, i.e., the standard deviation of the Gaussian function. LoG(*i*, *j*, *σ*) is the discrete LoG kernel defined by [45]:

(3) $$LoG\left(i,j,\sigma \right)=\frac{i^{2}{+j}^{2}-{2\sigma }^{2}}{2\pi \sigma^{6}}\cdot exp\left(-\frac{i^{2}+j^{2}}{2\sigma^{2}}\right)$$

The edges of large-scale impact craters are generally more profound and extensive, accompanied by lower spatial eigen frequencies, whereas our most interesting meter-sized impact craters manifest as more superficial and slimmer in appearance, with higher spatial eigen frequencies. Therefore, we converted the PHS image into frequency domain maps via Fourier transformation, in which the low-frequency data are located in the central regions and high-frequency specifics situated in marginal areas. In the frequency domain map, we only permit the eigen frequency associated with the meter-sized impact crater to pass through. The Discrete Fourier Transform (DFT) is defined as Equation (4) [47].

(4) $$F\left(u,v\right)=\frac{1}{W_{img}\times H_{img}}\sum_{y=0}^{H_{img}-1}\left[\sum_{x=0}^{W_{img}-1}M\left(x,y\right)exp\left(\frac{-i\times 2\pi ux}{W_{img}}\right)\right]exp\left(\frac{-i\times 2\pi vy}{H_{img}}\right)$$

where (*u*, *v*) are the coordinates on the frequency domain map. *W_img_* and *H_img_* are the width and height of the PHS image, respectively. The frequency domain map reserves the same size as the images prior to the Fourier transform. Subsequently, the region *A* that corresponds to the eigen frequency of meter-sized impact craters on the frequency domain map *F*(*u*, *v*) will be identified by further experiment (Section 3.1). Therefore, a band-pass mask *U*(*u*, *v*) is designed to selectively transmit information corresponding to the eigen frequency range of meter-sized impact craters in the spatial frequency domain, as described by the following equation.

(5) $$U\left(u,v\right)=\left\{\;\begin{array}{ll} 1 & when\;\left(u,v\right)\in A\\ 0 & others \end{array}\right.$$

The mask *U*(*u*, *v*) is applied to *F*(*u*, *v*) before the inverse discrete Fourier transform:

(6) $$R_{0}\left(x,y\right)=\sum_{u=0}^{W_{img}-1}\left[\sum_{v=0}^{H_{img}-1}\left[F\left(u,v\right)\times U\left(u,v\right)\right]exp\left(\frac{i\times 2\pi vy}{H_{img}}\right)\right]exp\left(\frac{i\times 2\pi ux}{W_{img}}\right)$$

The frequency domain bandpass filtered image *R*_0_ is generated by Equation (6), which characterizes the meter-sized impact craters. The bandpass filtered map based on 2D-DFT and its inverse transformation provide a novel pseudo-spectrum distinct from traditional edge detection methods. This highlights the scale characteristics of meter-sized impact craters more effectively.

Therefore, we obtained three pseudo-spectra for the meter-sized impact craters, which are the extracted features from the illumination incidence direction, isotropic second-order differential, and eigen frequency, respectively. These three extraction modalities are then amalgamated to construct a PMS image ([*R*_0_ *G*_0_ *B*_0_]^T^). As the PMS image only contains three pseudo-spectra with different extracted features, other details from the original image are lost. Thus, in the subsequent panchromatic sharpening operation, the PMS image is fused with the PHS image to form the PSEF image, as shown in Figure 2. In this procedure, the PMS image ([*R*_0_ *G*_0_ *B*_0_]^T^) is converted into the Hue-Saturation-Intensity (HSI) space [*I*_0_ *t*_10_ *t*_20_]^T^ using the subsequent Equation (7).

(7) $$\left[\begin{array}{c} I_{0}\\ t_{10}\\ t_{20} \end{array}\right]=\left[\begin{array}{ccc} 1/3 & 1/3 & 1/3\\ -\sqrt{2}/6 & -\sqrt{2}/6 & 2\sqrt{2}/6\\ 1/\sqrt{2} & -1/\sqrt{2} & 0 \end{array}\right]\left[\begin{array}{c} R_{0}\\ G_{0}\\ B_{0} \end{array}\right]$$

It is imperative to recognize that the auxiliary parameters *t*_10_ and *t*_20_ exhibit a defined mathematical correlation with hue *H* and saturation *S*, as illustrated by the following equations:

(8) $$\left\{\begin{array}{c} H=\tan^{-1}\left(\frac{t_{20}}{t_{10}}\right)\\ S=\sqrt{{t_{10}}^{2}+{t_{20}}^{2}} \end{array}\right.$$

Subsequently, *I*_0_ is substituted with the grayscale of the PHS image (*M*), followed by a transformation of the updated HSI space image [*M t*_10_ *t*_20_]^T^ back into the red-green-blue (RGB) space utilizing the equation:

(9) $$\left[\begin{array}{c} R_{PSEF}\\ G_{PSEF}\\ B_{PSEF} \end{array}\right]=\left[\begin{array}{ccc} 1 & -1/\sqrt{2} & 1/\sqrt{2}\\ 1 & -1/\sqrt{2} & -1/\sqrt{2}\\ 1 & \sqrt{2} & 0 \end{array}\right]\left[\begin{array}{c} M\\ t_{10}\\ t_{20} \end{array}\right]$$

Consequently, we acquired the PSEF image [*R_PSEF_ G_PSEF_ B_PSEF_*]^T^.

All slices are converted to form a PSEF dataset with manually annotated labels in Section 2.1, which includes the training, validation, and test datasets. The training dataset and partial validation dataset from the PSEF dataset are fed into the YOLOv5 framework to train the PSEF model, which is shown in Figure 2. The crater detection model in this work is developed from the YOLOv5 framework, which is a single-stage object detection architecture composed of several key components [48,49]. Firstly, the input images are processed by the Backbone module, which creates a rich hierarchical representation of the visual data by identifying various features at different levels of abstraction. Next, the Neck module further processes these features, enhancing the ability to detect the objects of the model at various scales through top-down and bottom-up pathways. Finally, the Head module predicts the bounding boxes and class probabilities for the detected object using anchor boxes.

As shown in Figure 2, we can send PHS images or PSEF images as the input images for the YOLOv5 framework. As lots of deep-learning-based object detection networks are designed for color images (three-channel and multi-modality), the PHS images generate Basal images with identical values in three channels through the self-stacking method, that is, the Basal images have three channels with identical values, i.e., single-modality. Consequently, using single-channel images or three-channel single-modality PHS images as inputs only provides insufficient information. While PSEF images are color images with three channels possessing different values, which represent three distinct modalities that are specifically enhanced to detect meter-sized impact craters, the new proposed PSEF images can therefore address the limitations of single-modality images inputted in neural networks via enriching the features of the SOD.

### 2.3. Evaluation Metrics

Precision (*P*) and recall (*R*) are adopted firstly to evaluate the accuracy of our automatic CDA and further refine the hyperparameters of our crater extraction and post-processing procedures [50]. *P* quantifies the exactitude of the algorithm in identifying impact craters, while *R* measures the completeness of that. The equations to calculate these metrics are as follows:

(10) $$P=\frac{TP}{TP+FP}\times 100\%$$

(11) $$R=\frac{TP}{TP+FN}\times 100\%$$

where *TP* is the true positives quantity, referring to the predicted bounding boxes that align with the ground truth. *FP* is the false positives quantity, indicating the predicted bounding boxes that diverge from the ground truth. *FN* is the false negatives quantity, describing the ground truth bounding boxes overlooked by the model. We also introduced two other metrics, i.e., the *F*_1_-score and mean average precision (*mAP*), to comprehensively represent the average values of *P* and *R*. The *F*_1_-scores are the harmonic mean of *P* and *R*. *mAP* quantifies the area under the *P-R* curve averaged across different classes.

(12) $$F_{1}-score=\frac{2PR}{P+R}\times 100\%$$

(13) $$mAP=\frac{1}{\left|C\right|}\sum_{c\in C}\left(\frac{1}{\left|K\right|}\sum_{k\in K}\left(\int_{0}^{1}P\left(c,k,R\right)dR\right)\right)$$

where C is the set of detected target classes, and K is the set of intersection over union (IoU) thresholds, which quantifies the ratio between the overlap of the detected bounding boxes and the ground truth boxes and their union. The vertical bars represent the cardinality of the set. The integral term denotes the area under the *P–R* curve for a specific class *c* and an IoU threshold *k*. For the metric *mAP*@50, K = [0.50], while for the metric *mAP*@50-95, K ranges from 0.50 to 0.95 in increments of 0.05. During the validation phase in this study, we employed the metrics *mAP*@50 and *mAP*@50-95 for the single class of meter-sized impact craters, while the *F*_1_*-score* is adopted as the evaluative benchmark to ascertain the detection accuracy of the model in the post-processing parameter optimization phase.

We should note that these aforementioned evaluation metrics are derived from the proportion of true positives. To examine the consistency between these predicted craters and the labelled craters more precisely, we introduced three extra metrics that represent the consistency in size, shape, and location between the predicted and labelled impact crater.

The first metric is the diameter relative error *δ_D_*, which denotes the size discrepancy between a true positive predicted bounding box and its manually annotated ground truth counterpart [12].

(14) $$\delta_{Di}=\frac{D_{TPi}-D_{GTi}}{D_{GTi}}\times 100\%=\left(\frac{W_{TPi}+H_{TPi}}{W_{GTi}+H_{GTi}}-1\right)\times 100\%$$

where *D_TP_*, *W_TP_*, and *H_TP_* are the diameter, width, and height of a true positive predicted crater bounding box, respectively. While *D_G__T_*, *W_G__T_*, and *H_G__T_* are the diameter, width, and height of the corresponding ground truth crater bounding box, respectively. The subscript *i* denotes the *i*th craters. The diameter *D* is approximated by the arithmetic mean of *W* and *H* within the bounding box of the crater.

Impact craters can be considered as circular targets theoretically, but they are not perfect circles in reality. Therefore, impact craters have been modelled as conic sections with an eccentricity less than 1 [51,52]. Thus, the variation in eccentricity, i.e., the eccentricity error Δ*e*, is employed in this work to assess the shape difference between the predicted and labelled impact craters, which is defined as follows:

(15) $$\Delta e=e_{TPi}-e_{GTi}=\sqrt{1-\frac{b_{TPi}^{2}}{a_{TPi}^{2}}}-\sqrt{1-\frac{b_{GTi}^{2}}{a_{GTi}^{2}}}$$

where *e* represents the eccentricity of the crater modelled as a conic section. The parameter *a* is the length of the semi-major axis, while *b* is that of the semi-minor axis.

The third metric is the location error Δ*L*, which equals the distance between the center of a true positive crater and that of its corresponding ground truth, thereby representing the positional discrepancy:

(16) $$\Delta L=\sqrt{{\left(x_{0TPi}-x_{0GTi}\right)}^{2}+{\left(y_{0TPi}-y_{0GTi}\right)}^{2}}$$

where *x*_0_ and *y*_0_ represent the central coordinates of the crater.

The metrics *δ_D_*, Δ*e*, and Δ*L* represent the deviations in size, shape, and location between a true positive predicted crater and its ground truth, respectively. The larger these metrics are, the greater the discrepancies between the true positives and ground truths. By calculating and statistically analyzing these metrics for the true positives by an automatic CDA, we can evaluate the precision of the impact crater detection algorithm. Further error analysis experiments will be discussed in Section 3.4.

## 3. Results

### 3.1. Feature Extraction and Enhanced Fusion

**Figure 3:**
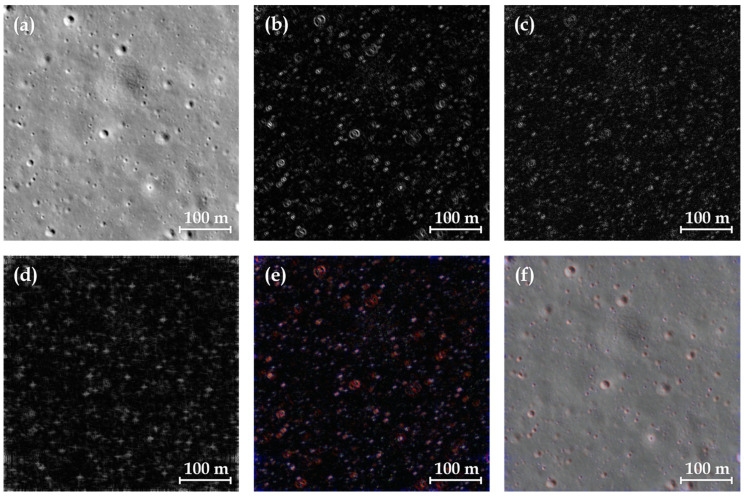
Illustration of images in the pseudo-spectral space. (**a**) The original PHS image. (**b**) Incidence features of crater rims obtained by the Sobel operator along the direction of incident light on the PHS image. (**c**) Isotropic features of meter-sized craters obtained by the LoG operator on the PHS image. (**d**) Eigen frequency features of meter-sized craters obtained by the bandpass filter in the frequency domain on the PHS image. (**e**) The PMS image obtained by amalgamating the images (**b**–**d**). (**f**) The PSEF image obtained by panchromatic sharpening on the PMS and PHS images.

Adopting the DOM from LRO-NAC with ID M1303619844, i.e., the remote sensing image over the CE-4 landing site with the light incidence angle value of 0.74 radians, we constructed the PSEF images using the methods described above. The original PHS image is presented in Figure 3a, and then the Sobel operators with a three-pixel kernel size are applied to this PHS image in both the *x* and *y* directions to generate the edge feature maps. These maps are subsequently weighted and combined according to the cosine and sine values of the incident light angle. The processes involving the Sobel operator and the combination are performed in 16-bit format, while the final images are saved in 8-bit format, as depicted in Figure 3b. A LoG operator with a convolution kernel size of three pixels is also applied to the PHS images, and the resulting isotropic features are shown in Figure 3c. Similarly, the intermediate process of LoG operator processing is conducted in 16-bit format but saved in 8-bit.

**Figure 4:**
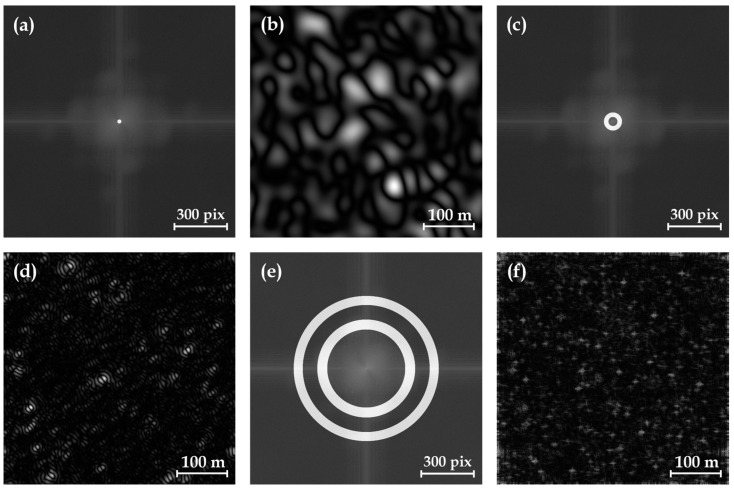
Frequency domain maps of PHS images with various mask regions. (**a**) Only the central 1–11 pixels annular mask region in the frequency domain of the PHS image is allowed to pass through. (**b**) The pseudo-spectrum obtained after the inverse DFT exhibiting eigen frequency features of impact craters with diameters in the hundreds of meters. (**c**) The annular mask region of 25–50 pixels in the frequency domain. (**d**) Eigen frequency features of impact craters with diameters over 20 m. (**e**) The concentric dual annular masks of 215–270 pixels and 350–400 pixels in the frequency domain. (**f**) Eigen frequency features for meter-sized impact craters.

Within the frequency domain image created from the PHS image through DFT, a bandpass mask is employed to extract the domain information on meter-sized impact craters. As discussed above, the center of the frequency domain image is dominated by low spatial frequency feature information, while high spatial frequency feature information is distributed in the marginal area. In the frequency domain image shown in Figure 4a,b, the 1–11 pixels region around the center corresponds to the eigen frequencies of impact craters over 100 m in diameter, while the 25–50 pixels region corresponds to those of impact craters over 20 m in diameter, as shown in Figure 4c,d. In our frequency domain bandpass filtering, the annular mask is the region that allows information to pass through, thereby enabling the extraction of corresponding features through inverse Fourier transformation. We conducted extensive experiments to adjust different masks to find the frequencies of impact craters at various positions in the frequency domain image. We found that the concentric annular regions of 215–270 pixels and 350–400 pixels are the optimal bandpass filter masks to extract the eigen frequency features of meter-sized impact craters, as shown in Figure 4e,f. Using this specific mask, the eigen frequency features of meter-sized craters are expressed, while information from impact craters of other sizes is suppressed.

After those procedures, we amalgamated these three pseudo-spectra to produce the PMS image in the pseudo-spectral space, which is shown in Figure 3e. Moreover, to complete the lost detailed information in the PMS image, we performed the panchromatic sharpening method to generate the PSEF image through enhanced fusion of the PMS and PHS images, as shown in Figure 3f.

### 3.2. Training

Datasets constructed using these PSEF images are also labelled as described in Section 2.1 and then are input into YOLO networks to train the PSEF model. Fives slices from the validation dataset are used in conjunction with the training dataset for learning within the YOLO framework. The remaining 10 slices from the validation dataset will be used to optimize the post-processing parameters in Section 3.3. Using the Basal image as the input, we also constructed a control group with all processing methods identical to those in the PSEF model.

The training process is conducted using two RTX 3090 GPUs, each with 24 GB of CUDA memory and a 36 vCPU AMD EPYC 9754 128-Core Processor. Training is carried out over 500 epochs with a batch size of 64 and 32 worker processes, and the optimizer is Stochastic Gradient Descent (SGD).

The loss functions of the training processes include three components, i.e., the box loss, the object loss (also known as confidence loss), and the class loss. The box loss is used to measure the prediction on the position and size of the bounding boxes. The object loss quantifies the confidence in the presence of a target within a given bounding box. If there is a target in the bounding box, the confidence is 1. Otherwise, it should be near 0. Thus, the object loss function contributes to distinguishing targets from the background. It should be noted that in this study, the meter-sized impact crater dataset we focused on is characterized by sparse features, a dense distribution, uniform background, and few negative examples. Therefore, we assigned a higher weight to the object loss. The box loss weight is set as 0.05, while the object loss weight is set as 10.0. As there is only one category of detection target in this work, i.e., impact crater, the class loss is thus maintained at 0.

The results in the validation set for the best epoch during the training process are shown in Table 1. The *P* values acquired from the Basal model and the PSEF model are 0.912 and 0.943, respectively, indicating the better precision performance by the PSEF model. *R* measures the proportion of true positives among all ground truths. In the best epoch, the *R* values of the Basal model and PSEF model are 0.806 and 0.891, respectively. This demonstrates the enhanced effectiveness of the PSEF model in correctly identifying the real positive cases. During the training process, the automatically generated results for *P* and *R* may not always be balanced well [53]. Thus, *mAP* is a key metric to evaluate the performance of detection models. The *mAP*@50 and *mAP*@50-95 of the PSEF model are 0.937 and 0.658, respectively, which are better than those of the Basal model.

### 3.3. Optimization of Post-Processing Parameters

The dataset of meter-sized craters used in this study is characterized by small scale, large sample number, and significant degradation in some cases. When the YOLO framework is employed for such detection, it tends to produce several candidate bounding boxes overlapping frequently and significantly for one target. Therefore, NMS is introduced as a crucial post-processing step to address this question; NMS is an essential technique to refine the output of object detection models [54]. NMS reduces redundancy among these boxes, ensuring each detected object is represented by only one bounding box, which is measured by the principal parameters of the confidence threshold (*σ*) and the IoU threshold (*τ*).

**Figure 5:**
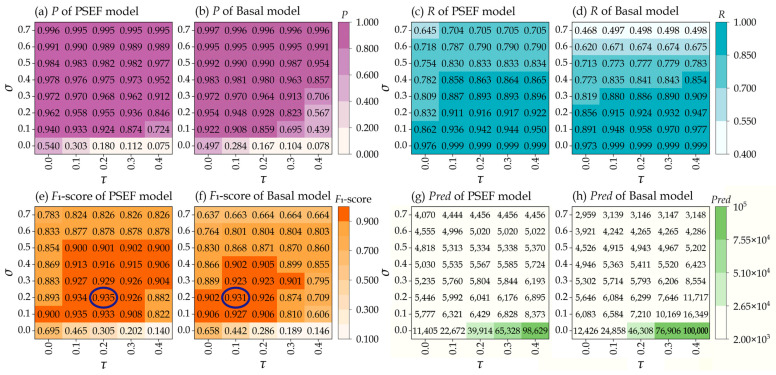
Illustrations for the optimization of the confidence threshold (*σ*) and IoU threshold (*τ*) using the discrete exhaustive search (DES) method. The *F*_1_-score in the best post-processing parameters for the PSEF model and the Basal model are highlighted with blue circles.

Initially, all detected bounding boxes with a confidence above *σ* are sorted in descending order based on their confidence. Starting with the box possessing the highest confidence, the IoU is calculated between this box and all the others. If the IoU exceeds a predefined threshold (*τ*), the overlapping boxes are suppressed. This process is repeated for each box in the sorted list. The iteration stops when all boxes have either been identified as the final detections or suppressed. After that, ten images from the validation dataset are used to optimize the aforementioned two post-processing parameters, i.e., the confidence threshold and the IoU threshold. The optimizing post-processing parameters for the Basal model and the PSEF model are acquired through the discrete exhaustive search (DES) method [6] in which the optimizing post-processing parameters *σ* and *τ* are taken as *σ* ∈ {0, 0.1, 0.2, 0.3, 0.4, 0.5, 0.6, 0.7} and *τ* ∈ {0, 0.1, 0.2, 0.3, 0.4}, respectively. The *F*_1_*-score* serves as a key evaluation metric for the detection capability of the model with respect to the target under the specified post-processing parameter settings. The results are displayed in Figure 5, in which the maximum number of predictions per slices during detection is set to 10,000.

As *τ* increases from 0 to 0.4, the number of predicted bounding boxes (*Pred*) rises sharply. However, this increase mainly originates from the duplicate boxes classified as false positives, thereby reducing the *P*. That is, the increase in *τ* diminishes the effectiveness of suppressing larger overlaps in repeated prediction boxes. However, as *σ* increases from 0 to 0.7, *Pred* gradually decreases. The model only exhibits the prediction boxes with high confidence values. Consequently, the model outputs fewer but more accurate prediction boxes. However, true positives may be discarded mistakenly for not meeting the higher threshold, which reduces *R* and thus the *F*_1_-score.

Compared with the Basal model, the PSEF model’s *Pred* decreases more gently as *τ* decreases. For instance, with *σ* = 0.1, when *τ* decreases from 0.4 to 0.3, the *Pred* of the PSEF model decreases by 1565, which is lower than the value of 6180 for the Basal model. With σ = 0.2 and the decrease in τ from 0.4 to 0.3, the decrease in *Pred* is 719 for the PSEF model and 4071 for the Basal model. These results suggest that the PSEF model achieves greater accuracy in edge detection. With higher *τ* settings, detection boxes are considered duplicates only when their overlap is substantial, thereby retaining more exactly positioned boxes. Greater precision in edge detection enables the model to distinguish closely adjacent and slightly overlapping targets more accurately. This accuracy arises from the detection boxes possessing more accurate positions and shapes of the targets. Conversely, even with higher *τ* settings, a model with worse precision in edge detection may mistakenly merge distinct targets due to less accurate detection boundaries. At higher *τ* values, the PSEF model maintains a lower *Pred*, possibly due to the superior edge detection precision. This enables it to effectively distinguish and retain accurate detection boxes during the NMS process, thereby minimizing false suppression and enhancing the overall detection accuracy for impact craters.

The PSEF model records its highest *F*_1_-score of 0.935 with *σ* = 0.2 and *τ* = 0.2, while the Basal model achieves its max *F*_1_-score of 0.931 with *σ* = 0.2 and *τ* = 0.1. Although the *F*_1_-scores of the PSEF and Basal models are close in their optimal post-processing parameters, the PSEF model maintains higher *F*_1_-scores with a broader range of *σ* and *τ* parameters. Among our 40 sampling results, the PSEF model achieves *F*_1_-scores exceeding 0.9 in 19 parameter combinations, while the Basal model achieves this value in only 11 parameter combinations. This demonstrates the stronger robustness of the PSEF model to variations in NMS parameter settings. Consequently, employing the PSEF model to detect impact craters in new regions without annotations tends to yield superior results. That is, when the PSEF model is adopted to detect the new unknown regions, although specific adjustment of the NMS parameters is not feasible, it maintains better performance across a wider range of settings. The robustness to the NMS parameter settings is essential to detect the numerous and densely distributed meter-sized impact craters. Therefore, the PSEF model is more practical than the Basal model for detecting meter-sized impact craters in unknown regions.

### 3.4. Error Analysis of True Positives

**Figure 6:**
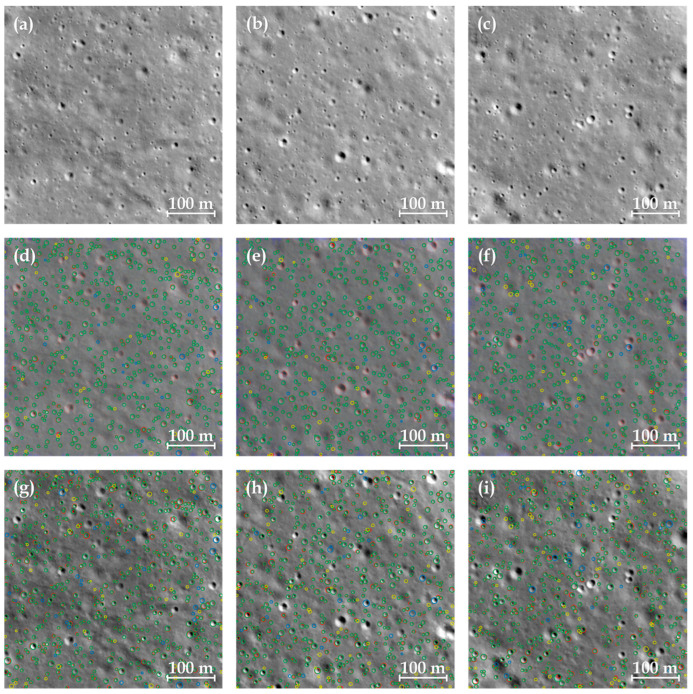
Visualization of partial test dataset slices and their corresponding automatic identification results. (**a**–**c**) are images from the test dataset. (**d**–**f**) are the identification results from the PSEF model. (**g**–**i**) display the identification results of the Basal model. Among them, the green, red, yellow, and blue circles refer to true positives, ground truth, false negatives, and false positives, respectively.

The results in Section 3.3 indicated that with the optimized post-processing parameters, the PSEF model exhibits obvious advantages in *P*, *R*, and *F*_1_-scores in the 10 slices from the validation dataset compared to the Basal model. We compared the performances of the PSEF model and the Basal model on the test dataset, each with their respective optimal post-processing parameters, and listed several metrics in Table 1. The results from Table 1 show that the performance of the PSEF model surpasses that of the Basal model. There are relatively few CDAs focusing specifically on meter-sized craters. Moreover, compared to other advanced CDAs developed for tens or hundreds of meters in diameter, our PSEF model shows obvious advantages in the *P*, *R*, and *F*_1_-score metrics, as shown in Table S1. As is discussed in the previous section, the slighter decrease in *Pred* with the reduction in *τ* for the PSEF model indicates a superior recognition effect on the edges of impact craters. Preliminary observations from Figure 6 suggest that the true positives bounding box matches the corresponding ground truth better in the PSEF model than that of the Basal model.

**Figure 7:**
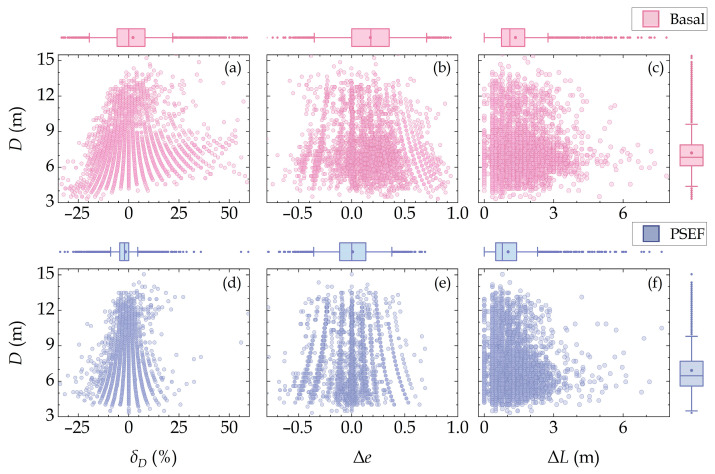
Statistical marginal distribution of the diameter relative error (*δ_D_*), the eccentricity error (Δ*e*), and the location error (Δ*L*) between true positives and the corresponding ground truths in the test dataset under the optimal post-processing parameters for the Basal model (**a**–**c**) and the PSEF model (**d**–**f**). The scatter points represent the distribution of error metrics—diameter for each true positive. The top and bottom of each box plot represent 75% and 25% of the data, respectively. Within the box, the solid line indicates the median, while the dot denotes the mean value. The whiskers extending from the boxes represent the interval within one standard deviation of the mean. Outliers that exceed the whiskers are marked individually.

Subsequent analyses involving the diameter relative error (*δ_D_*), the eccentricity error (Δ*e*), and the location error (Δ*L*) for each true positive and their ground truth in the PSEF and Basal models are performed to further quantify the accuracy of the prediction box edges. The marginal distribution diagrams of these three error metrics for all true positives in the test dataset of both the PSEF and Basal models are depicted in Figure 7. To determine which model demonstrates an error distribution closer to zero, statistical values of the error metrics, including the mean, median, etc., are also calculated accordingly, which are presented alongside the first three error metrics in Table 2.

From the test dataset shown in Table 2, we found that the mean and median values of *|δ_D_|*, *|*Δ*e|*, and Δ*L* acquired from the PSEF model are notably closer to zero than those from the Basal model, suggesting the reduced recognition errors in the size, shape, and location of impact craters by the PSEF model. Moreover, the standard deviations and the *IQRs* of *δ_D_,* Δ*e*, and Δ*L* serve as metrics to measure the consistency of the edge detection of the model. The standard deviations and IQR values for *δ_D_*, Δ*e*, and Δ*L* in the PSEF model are lower than those in the Basal model, indicating the improved consistency in the edge detection of the PSEF model. Figure 7 demonstrates that the statistical marginal distributions of *δ_D_*, Δ*e*, and Δ*L* from the PSEF model are closer to zero, and the scatter points are more concentrated. There results demonstrate that compared to the Basal model, the PSEF model shows less error during the detection of meter-sized craters, thus achieving more reliable and accurate edge recognition results. The smaller *δ_D_* of the PSEF model is possibly due to its eigen frequency features effectively capturing the scale information of meter-sized impact craters, while the smaller Δ*e* and Δ*L* may be attributed to the isotropic features and illumination incidence features effectively extracting the edge variations of impact craters.

## 4. Conclusions

We proposed a new pseudo-spectral spatial feature extraction and enhanced fusion (PSEF) image input method to detect meter-sized impact craters on the lunar surface by the YOLOv5 framework, which addresses the difficulty in detecting feature scarce small impact craters using deep learning methods. Adopting the DOM of the LRO-NAC image captured on the CE-4 landing site, the illumination incidence features, isotropic features, and eigen frequency features are extracted by means of Sobel filtering, Laplacian of Gaussian (LoG) filtering, and frequency domain bandpass filtering, respectively. Then, the PSEF images are created by pseudo-spectral spatial techniques to preserve additional details from the original DOM data. This PSEF image dataset is input into the YOLOv5 framework to train the PSEF model. Compared with the model trained by the self-stacking PHS images composed of the Basal images, the detection results show that this new PSEF method significantly improves the detection accuracy of meter-sized craters.

After that, two post-processing parameters, i.e., the confidence threshold (*σ*) and IoU threshold (*τ*), are optimized using the validation dataset and DES method. We found that although the *F*_1_-scores of the PSEF and Basal models are close in their optimal post-processing parameters, the PSEF model maintains higher *F*_1_-scores with a broader range of *σ* and *τ* parameters. The stronger robustness of the PSEF model makes it more practical than the Basal model to detect meter-sized impact craters in unknown regions.

Our PSEF model achieves a *P* of 0.943, *R* of 0.891, *F*_1_-score of 0.916, and *mAP*@50 of 0.937 on the validation dataset. In the test dataset, the PSEF model maintains a high *P* of 0.969, *R* of 0.932, and *F*_1_-score of 0.950, demonstrating superior robustness and accuracy in practical deployment. In addition to these common evaluation metrics, we designed several metrics to examine the detection performance, including the diameter relative error, the eccentricity error, and the location error between each true positive and their ground truth. The PSEF model exhibited superior performance over the Basal model in predicting the size, shape (eccentricity), and position of impact craters. The error metrics of the PSEF model, including the mean, median, IQR, and standard deviation, are better than those of the Basal model, indicating its ability to predict features that are more consistent, stable, and closer to the true characteristics of impact craters.

Above all, the superior edge detection and feature extraction provided by the PSEF model contribute to more accurate and consistent crater identification, which can supply crucial and helpful support for subsequent applications in the evaluation of planetary resources, decoding their geological evolutionary histories, exploration missions, etc.

## Figures and Tables

**Table 1:** Evaluation metrics of PSEF and Basal models in both validation and test datasets.

Result	Model	*P*	*R*	*mAP*@50	*mAP*@50-95	*F*_1_ *-Score*
Best epoch results in validation dataset	PSEF	0.943	0.891	0.937	0.658	0.916
	Basal	0.912	0.806	0.898	0.498	0.856
Optimal post-processing parameter results in test dataset	PSEF	0.969	0.932	/	/	0.950
	Basal	0.951	0.886	/	/	0.917

**Table 2:** The mean, median, interquartile range (IQR), and standard deviations for the error metrics including the diameter relative error (*δ_D_*), absolute diameter relative error (|*δ_D_*|), eccentricity error (Δ*e*), absolute eccentricity error (|Δ*e*|), and location deviation (Δ*L*) for both the PSEF and Basal models in the test dataset.

Model	Analytical Scale	*δ_D_* (%)	|*δ_D_*| (%)	Δ*e*	|Δ*e*|	Δ*L* (m)
PSEF	mean	/	3.94	/	0.168	1.03
	median	/	3.03	/	0.124	0.78
	IQR	4.54	/	0.246	/	0.91
	standard deviation	5.67	/	0.233	/	0.73
Basal	mean	/	8.91	/	0.258	1.35
	median	/	6.52	/	0.250	1.11
	IQR	13.76	/	0.353	/	1.00
	standard deviation	12.26	/	0.275	/	0.94

## Data Availability

Data are contained within the article.

## Supplementary Materials

The following supporting information can be downloaded at: https://www.mdpi.com/article/10.3390/s24165206/s1, Table S1: Comparison of Key Metrics for Advanced Automatic Impact Crater Detection Algorithms.

## References

[1] Ashley J.W., Robinson M.S., Hawke B.R., van der Bogert C.H., Hiesinger H., Sato H., Speyerer E.J., Enns A.C., Wagner R.V., Young K.E. (2012). Geology of the King crater region: New insights into impact melt dynamics on the Moon. J. Geophys. Res. -Planets.

[2] Dhingra D., Head J.W., Pieters C.M. (2017). Geological mapping of impact melt deposits at lunar complex craters Jackson and Tycho: Morphologic and topographic diversity and relation to the cratering process. Icarus.

[3] Bray V.J., Tornabene L.L., Keszthelyi L.P., McEwen A.S., Hawke B.R., Giguere T.A., Kattenhorn S.A., Garry W.B., Rizk B., Caudill C.M. (2010). New insight into lunar impact melt mobility from the LRO camera. Geophys. Res. Lett..

[4] Crawford I.A. (2015). Lunar resources: A review. Prog. Phys. Geogr..

[5] Yue Z.Y., Shi K., Di K.C., Lin Y.T., Gou S. (2023). Progresses and prospects of impact crater studies. Sci. China-Earth Sci..

[6] Liu J., Zeng X., Li C., Ren X., Yan W., Tan X., Zhang X., Chen W., Zuo W., Liu Y. (2020). Landing Site Selection and Overview of China’s Lunar Landing Missions. Space Sci. Rev..

[7] Sutoh M., Otsuki M., Wakabayashi S., Hoshino T., Hashimoto T. (2015). The Right Path: Comprehensive Path Planning for Lunar Exploration Rovers. IEEE Robot. Autom. Mag..

[8] Yu X.Q., Wang P., Zhang Z.X. (2021). Learning-Based End-to-End Path Planning for Lunar Rovers with Safety Constraints. Sensors.

[9] Menon M.S., Kothandhapani A., Sundaram N.S., Raghavan V., Nagaraj S. Terrain-based Analysis as a Design and Planning Tool for Operations of a Lunar Exploration Rover for the TeamIndus Lunar Mission. Proceedings of the 2018 SpaceOps Conference.

[10] Hu T., Yang Z., Kang Z.Z., Lin H.Y., Zhong J., Zhang D.Y., Cao Y.M., Geng H.M. (2022). Population of Degrading Small Impact Craters in the Chang’E-4 Landing Area Using Descent and Ground Images. Remote Sens..

[11] Robbins S.J. (2019). A New Global Database of Lunar Impact Craters &gt;1-2km: 1. Crater Locations and Sizes, Comparisons With Published Databases, and Global Analysis. J. Geophys. Res. -Planets.

[12] Fairweather J.H., Lagain A., Servis K., Benedix G.K., Kumar S.S., Bland P.A. (2022). Automatic Mapping of Small Lunar Impact Craters Using LRO-NAC Images. Earth Space Sci..

[13] Yang C., Zhao H.S., Bruzzone L., Benediktsson J.A., Liang Y.C., Liu B., Zeng X.G., Guan R.C., Li C.L., Ouyang Z.Y. (2020). Lunar impact crater identification and age estimation with Chang’E data by deep and transfer learning. Nat. Commun..

[14] Juntao Y., Shuowei Z., Lin L., Zhizhong K., Yuechao M. (2024). Topographic knowledge-aware network for automatic small-scale impact crater detection from lunar digital elevation models. Int. J. Appl. Earth Obs. Geoinf..

[15] Bugiolacchi R., Wöhler C. (2020). Small craters population as a useful geological investigative tool: Apollo 17 region as a case study. Icarus.

[16] Xiao Z.Y. (2016). Size-frequency distribution of different secondary crater populations: 1. Equilibrium caused by secondary impacts. J. Geophys. Res. -Planets.

[17] Kawashima O., Morota T., Ohtake M., Kasahara S. (2022). Size-frequency measurements of meter-sized craters and boulders in the lunar polar regions for landing-site selections of future lunar polar missions. Icarus.

[18] Cadogan P.H. (2020). Automated precision counting of very small craters at lunar landing sites. Icarus.

[19] Feng Q.H., Xu X.Z., Wang Z.X. (2023). Deep learning-based small object detection: A survey. Math. Biosci. Eng..

[20] Liu Y., Sun P., Wergeles N., Shang Y. (2021). A survey and performance evaluation of deep learning methods for small object detection. Expert Syst. Appl..

[21] Wu J.Q., Xu S.B. (2021). From Point to Region: Accurate and Efficient Hierarchical Small Object Detection in Low-Resolution Remote Sensing Images. Remote Sens..

[22] Tong K., Wu Y.Q. (2022). Deep learning-based detection from the perspective of small or tiny objects: A survey. Image Vis. Comput..

[23] Lang X., Yuan L., Li S., Liu M. (2024). Pipeline Multipoint Leakage Detection Method Based on KKL-MSCNN. IEEE Sens. J..

[24] Bo Z., Di K.C., Liu Z.Q., Yue Z.Y., Liu J., Shi K. (2022). A catalogue of meter-scale impact craters in the Chang’e-5 landing area measured from centimeter-resolution descent imagery. Icarus.

[25] La Grassa R., Cremonese G., Gallo I., Re C., Martellato E. (2023). YOLOLens: A Deep Learning Model Based on Super-Resolution to Enhance the Crater Detection of the Planetary Surfaces. Remote Sens..

[26] Tewari A., Khanna N. (2024). Arbitrary Scale Super-Resolution Assisted Lunar Crater Detection in Satellite Images. arXiv.

[27] Yan Y., Qi D., Li C. (2019). Vision-based crater and rock detection using a cascade decision forest. IET Comput. Vis..

[28] Zang S., Mu L., Xian L., Zhang W. (2021). Semi-Supervised Deep Learning for Lunar Crater Detection Using CE-2 DOM. Remote Sens..

[29] Prete R.D., Saveriano A., Renga A. A Deep Learning-based Crater Detector for Autonomous Vision-Based Spacecraft Navigation. Proceedings of the 2022 IEEE 9th International Workshop on Metrology for AeroSpace (MetroAeroSpace).

[30] Chatterjee S., Chakraborty S., Nath A., Chowdhury P.R., Deshmukh B. Near-Real-Time Detection of Craters: A YOLO v5 Based Approach. Proceedings of the 2023 International Conference on Machine Intelligence for GeoAnalytics and Remote Sensing (MIGARS).

[31] Zhu L.L., Geng X., Li Z., Liu C. (2021). Improving YOLOv5 with Attention Mechanism for Detecting Boulders from Planetary Images. Remote Sens..

[32] Hari R.V.S., Ambalam R., Kumar B.R., Ibrahim M., Ponnusamy R. Yolo5-Based UAV Surveillance for Tiny Object Detection on Airport Runways. Proceedings of the 2023 International Conference on Data Science, Agents & Artificial Intelligence (ICDSAAI).

[33] Su Y., Tan W.X., Dong Y.F., Xu W., Huang P.P., Zhang J.X., Zhang D.K. (2024). Enhancing concealed object detection in Active Millimeter Wave Images using wavelet transform. Signal Process..

[34] Ji C., Zhang F., Huang X.B., Song Z.W., Hou W., Wang B.Y., Chen G.Y. (2024). STAE-YOLO: Intelligent detection algorithm for risk management of construction machinery intrusion on transmission lines based on visual perception. IET Gener. Transm. Distrib..

[35] Mu L.L., Xian L.A., Li L.H., Liu G., Chen M., Zhang W. (2023). YOLO-Crater Model for Small Crater Detection. Remote Sens..

[36] Kim J.R., Muller J.P., van Gasselt S., Morley J.G., Neukum G., Team H.C. (2005). Automated crater detection, a new tool for Mars cartography and chronology. Photogramm. Eng. Remote Sens..

[37] Saheba S.M., Upadhyaya T.K., Sharma R.K. (2016). Lunar surface crater topology generation using adaptive edge detection algorithm. IET Image Process..

[38] Zuo W., Li C.L., Yu L.J., Zhang Z.B., Wang R.W., Zeng X.G., Liu Y.X., Xiong Y.Y. (2019). Shadow-highlight feature matching automatic small crater recognition using high-resolution digital orthophoto map from Chang’E Missions. Acta Geochim..

[39] Robinson M.S., Brylow S.M., Tschimmel M., Humm D., Lawrence S.J., Thomas P.C., Denevi B.W., Bowman-Cisneros E., Zerr J., Ravine M.A. (2010). Lunar Reconnaissance Orbiter Camera (LROC) Instrument Overview. Space Sci. Rev..

[40] Speyerer E.J., Wagner R.V., Robinson M.S., Humm D.C., Becker K., Anderson J., Thomas P. (2012). IN-FLIGHT GEOMETRIC CALIBRATION OF THE LUNAR RECONNAISSANCE ORBITER CAMERA. Int. Arch. Photogramm. Remote Sens. Spatial Inf. Sci..

[41] Zaka S.S., Majeed M.N., Dawood H.J.V.I. (2022). Blind Image Deblurring Using Laplacian of Gaussian (LoG) Based Image Prior. Int. J. Innov. Sci. Technol..

[42] Zhang Y., Han X., Zhang H., Zhao L. Edge detection algorithm of image fusion based on improved Sobel operator. Proceedings of the 2017 IEEE 3rd Information Technology and Mechatronics Engineering Conference (ITOEC).

[43] Huang G.W., Zhang F. (2024). The fast computation of multi-angle discrete fractional Fourier transform. Signal Process..

[44] Liao T.L., Peng C.Y., Hou Y.Y. (2023). Application of multi-party computation and error correction with image enhancement and convolution neural networks based on cloud computing. IET Image Process..

[45] Ghosal S.K., Mandal J.K., Sarkar R. (2018). High payload image steganography based on Laplacian of Gaussian (LoG) edge detector. Multimed. Tools Appl..

[46] Gunn S.R. (1999). On the discrete representation of the Laplacian of Gaussian. Pattern Recognit..

[47] Winograd S. (1976). On computing the Discrete Fourier Transform. Proc. Natl. Acad. Sci. USA.

[48] Gao T.Y., Wushouer M., Tuerhong G. (2023). DMS-YOLOv5: A Decoupled Multi-Scale YOLOv5 Method for Small Object Detection. Appl. Sci..

[49] Xue J., Zheng Y.G., Dong C.L.Y., Wang P., Yasir M. (2022). Improved YOLOv5 network method for remote sensing image-based ground objects recognition. Soft Comput..

[50] Silburt A., Ali-Dib M., Zhu C.C., Jackson A., Valencia D., Kissin Y., Tamayo D., Menou K. (2019). Lunar crater identification via deep learning. Icarus.

[51] Andrews-Hanna J.C., Zuber M.T., Gibson R.L., Reimold W.U. (2010). Elliptical craters and basins on the terrestrial planets. Large Meteor. Impacts Planet. Evol. IV.

[52] Christian J.A., Derksen H., Watkins R. (2021). Lunar Crater Identification in Digital Images. J. Astronaut. Sci..

[53] Wilhelm T., Wöhler C., Ieee Comp S.O.C. Uncertainty Guided Recognition of Tiny Craters on the Moon. Proceedings of the 25th International Conference on Pattern Recognition (ICPR), Electr Network.

[54] Qiu S.H., Wen G.J., Deng Z.P., Liu J., Fan Y.X. (2018). Accurate non-maximum suppression for object detection in high-resolution remote sensing images. Remote Sens. Lett..

