# Supplementary material for: Pseudo-Spectral Spatial Feature Extraction and Enhanced Fusion Image for Efficient Meter-Sized Lunar Impact Crater Automatic Detection in Digital Orthophoto Map
Source: Sensors (Basel). 2024 Aug 11;24(16):5206. doi: 10.3390/s24165206 (PMC11360746; doi:10.3390/s24165206)
Supplement: Supplementary file 1 [file sensors-24-05206-s001.zip › sensors-3108939-supplementary.pdf]

sensors

MDPI

Supplementary materials

Table S1. Comparison of Key Metrics for Advanced Automatic Impact Crater Detection Algorithms

Model	Crater diameter / m	P	R	F_1-score
Fairweather et al. [12]	20 - 40	0.78	0.92	0.84
Zang et al. [28]	< 100	0.915	0.549	0.687
Mu et al. [35]	< 100	0.850	0.655	0.740
Grass et al. [25]	100 - 1000	0.899	0.872	0.885
PSEF *	< 14	0.969	0.932	0.950

* Model proposed in this paper
